# Photodynamic Diagnosis Using 5-Aminolevulinic Acid with a Novel Compact System and Chromaticity Analysis for the Detection of Oral Cancer and High-Risk Potentially Malignant Oral Disorders

**DOI:** 10.3390/diagnostics12071532

**Published:** 2022-06-23

**Authors:** Seiko Tatehara, Toru Sato, Yusuke Takebe, Momoka Fujinaga, Chiaki Tsutsumi-Arai, Yumi Ito, Kazuhito Satomura

**Affiliations:** 1 Department of Oral Medicine and Stomatology, School of Dental Medicine, Tsurumi University, 2-1-3 Tsurumi, Tsurumi-ku, Yokohama 230-8501, Japan; sato-tr@tsurumi-u.ac.jp (T.S.); takebe-yusuke@tsurumi-u.ac.jp (Y.T.); fujinaga-m@tsurumi-u.ac.jp (M.F.); tsutsumi-c@tsurumi-u.ac.jp (C.T.-A.); satomura-k@tsurumi-u.ac.jp (K.S.); 2 Division of Oral Pathology, Tsurumi University Dental Hospital, 2-1-3 Tsurumi, Tsurumi-ku, Yokohama 230-8501, Japan; ito-yu@tsurumi-u.ac.jp

**Keywords:** photodynamic diagnosis, oral cancer, oral epithelial dysplasia, oral potentially malignancy disorders, 5-aminolevulinic acid, protoporphyrin IX, fluorescence

## Abstract

Detecting early-stage oral cancer and precancerous lesions are critical to improving patient prognosis and quality of life after treatment. Photodynamic diagnosis using 5-aminolevulinic acid enables the detection of malignant lesions. This study aimed to improve the diagnostic accuracy of photodynamic diagnosis using an objective chromaticity analysis of fluorescence emitted from oral lesions. Sixty-seven patients with clinically suspicious oral cavity lesions underwent photodynamic diagnosis after topical application of 5-aminolevulinic acid solution, followed by imaging and histological evaluation of the lesions. Chromaticity red and green values were measured from the fluorescence images on the lesion, and the red-to-green ratio was calculated. The photodynamic diagnosis allowed for the visualization of oral cancer and high-risk dysplasia as red fluorescence. Compared to low-risk dysplasia and benign lesions, oral cancer and high-risk dysplasia areas had a significantly higher red value and red-to-green ratio. After setting the cutoff value, sensitivity and specificity were 83.3–88.7% and 83.3–83.9%, respectively, when discriminating between oral cancer or high-risk dysplasia and low-risk dysplasia or benign lesions. Photodynamic diagnosis combined with chromaticity analysis may be a valuable diagnostic tool for detecting oral lesions, with a high likelihood of malignant transformation.

## 1. Introduction

Oral cancer arising from the lip and oral cavity is the 16th most common cancer globally [1]. Despite a declining incidence of this cancer worldwide, countries with a high rate of tobacco use, alcohol consumption, or a high aging population are still experiencing an increase in oral cancer incidence [1,2]. Almost 355,000 lip and oral cavity cancer cases are diagnosed every year, with 177,000 deaths occurring in 2018 [1,2]. The overall 5-year survival rate is approximately 50%, which is worse than most other cancers, primarily because most cases are detected at an advanced stage [3]. Indeed, if oral cancers were mostly detected in early stages, the survival rate would be as high as 90%, with less oral dysfunction after treatment [4,5]. Unfortunately, cases with a locally advanced disease require a large resection and reconstruction, resulting in dysphagia, dysarthria, and low maintenance of the quality of life, including psychological distress and cosmetic disturbance. Thus, to diminish the resection area, alleviate dysphagia and dysarthria, and ultimately improve the survival rate, clinicians should strategically focus on detecting very early-stage oral cancer [6].

In 2017, the World Health Organization (WHO) defined oral potentially malignant disorders (OPMDs) as clinical presentations that carry a risk of cancer development in the oral cavity, whether in a clinically definable precursor lesion or in clinically normal oral mucosa [7]. OPMDs sometimes include histopathologically oral epithelial dysplasia (OED) [8], which is classified into three grades, namely, mild, moderate, and severe, based on the severity of the epithelial architectural and cytologic changes [7]. Previous reports have indicated that some dysplastic lesions undergo malignant transformation, and the transformation rate is 4.8–6% for mild, 15.7–18% for moderate, and 26.7–39% for severe OED [4,8,9]. In contrast, it is only 0.012% for non-dysplastic lesions [4,8,9]. From these results, most studies have indicated that OPMDs include severe and moderate OED that has a high malignant transformation rate, referred to as “high-risk OPMDs” [4,8,9,10]. Therefore, high-risk OPMDs should also be detected early to enable minimum intervention and lesion monitoring [10,11].

The conventional oral examination is used to diagnose oral mucosal lesions and is performed by inspection and palpation [12,13]. However, a conventional oral examination alone cannot detect very early-stage oral cancer and high-risk OPMDs or grade OED [4,14,15]. These lesions appear as superficial, minute, or minor lesions in normal mucosa, whereas frictional keratosis, leukoplakia, and leukoedema present clinically as high-risk OPMDs [16,17]. Therefore, there is an urgent need for easy, noninvasive, and accurate diagnostic aids [12].

Photodynamic diagnosis (PDD) using 5-aminolevulinic acid (ALA; ALA-PDD) enables the visualization of malignant lesions as red fluorescence [18,19,20]. ALA is an endogenous natural amino acid synthesized from glycine and succinyl coenzyme A in the mitochondria and is finally converted to heme through the biosynthetic heme pathway. After ALA administration, exogenous ALA is incorporated into the cell and converted to the fluorescent molecule protoporphyrin IX (PpIX) downstream of the biosynthetic pathway [21]. In normal cells, excess PpIX is quickly metabolized or released from the cells through regulation by negative feedback; however, PpIX accumulates at high levels in cancer cells [18,22,23]. Briefly, cancer cells with an accumulation of PpIX emit red fluorescence (635 nm) when irradiated with visible blue light (405 nm) [18]. Thus, ALA-PDD can be used to visualize cancers as red fluorescence. For the first time in the field of oral and maxillofacial surgery, Leunig et al. applied ALA-PDD to oral mucosa lesions and indicated that ALA-PDD had high sensitivity and low specificity for detecting oral cancer and severe to moderate dysplasia (90% and 60%, respectively) [24,25,26]. One cause of the low specificity might be the subjective judgment of whether the lesions emit red fluorescence; indeed, it is especially difficult to judge oral lesions with weak red fluorescence and depends greatly on the examiner’s experience and skills. Thus, some reports have attempted to qualify the fluorescence excited from the oral lesions [27,28,29,30] to improve specificity.

Consequently, there was an increase in the specificity of ALA-PDD for discriminating between oral cancer or dysplastic lesions and benign lesions. However, the PDD system and qualification method were too complicated for most dental clinics and hospitals to use because of the need for an endoscopic system, an instrument that emits 405-nm blue light, and spectroscopy for measuring the intensity of fluorescence. Therefore, a compact ALA-PDD device and a user-friendly qualification system are required. This study aimed to develop an innovative and handheld ALA-PDD device and estimate its utility. In addition, we sought to objectively improve the reliability of the fluorescent images using a simple chromaticity analysis for qualifying the fluorescence emitted by oral lesions.

## 2. Materials and Methods

### 2.1. Compact PDD System

**Figure 1:**
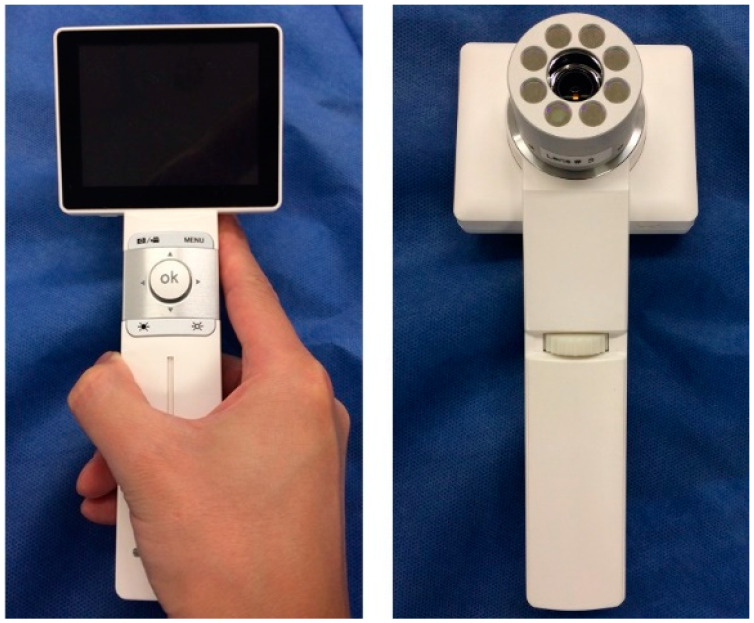
Novel handheld camera device for 5-aminolevulinic acid photodynamic diagnosis (ALA-PDD).

We developed a handheld, compact PDD system designed to irradiate lesions with visible blue light (405 nm) emitted by eight light-emitting diodes surrounding the imaging lens. Emission (EM), notch (NF), and infrared cut (IR) filters were used to detect red fluorescence (635 nm) emitted by the oral lesions. This system was equipped with a charge-coupled device camera, which was used to record the red fluorescent images (Figure 1).

### 2.2. Patients

Sixty-seven patients with clinically suspicious malignant lesions of the oral cavity underwent ALA-PDD. The patients visited the Department of Oral Medicine of Tsurumi University Dental Hospital between June 2014 and March 2019. The patients underwent a biopsy or a resection after ALA-PDD. The 80 lesion sites consisted of the tongue, oral floor, gingiva, and buccal mucosa (Table 1). Before we performed ALA-PDD, we explained the procedure to all patients using written instructions, and all patients provided written informed consent. Inclusion criteria included (1) age >20 years, (2) patients with lesions suspicious of oral cancer and undergoing a biopsy or resection for oral lesions, and (3) providing informed consent. Exclusion criteria included (1) patients with porphyria and photosensitivity, (2) patients with serious medical history (renal impairment or hepatic impairment), (3) patients with serious mental disorders, (4) pregnant women or nursing mothers, and (5) patients judged to be inappropriate to participate in this study by the examiners.

### 2.3. Examination Procedure

**Figure 2:**
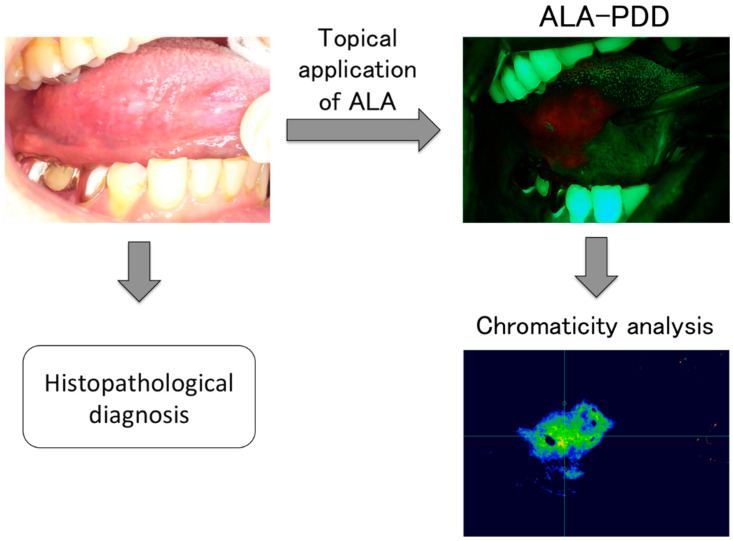
Observations of the oral cavity included the following steps: topical application of the ALA solution, observation using the PDD system, and histopathological diagnosis. Subsequently, the C_R_: chromaticity x (red) on the lesion sites was measured from the fluorescence images using Eye-Scale one software (I-system, Tokyo, Japan). The example shows a case with slight erosion of the tongue, which was diagnosed histopathologically as moderate oral epithelial dysplasia (OED). Images were converted by the software to measure C_R_.

First, the oral cavity was cleaned, including tartar control and tooth brushing as much as possible, and the ALA solution was applied topically to the lesions before PDD. Specifically, the gauze was soaked with 1% ALA hydrochloride solution, which was prepared by dissolving 50 mg of ALA hydrochloride powder (Cosmo Bio Co., Ltd., Tokyo, Japan) in 5 mL of water. The gauze was then incubated for 1–1.5 h with the lesions, including the healthy surrounding tissues, following a procedure modified from previous reports [24,25,26,27,28,29,30]. Patients were then asked to rinse their mouths. We then observed the lesions using the compact PDD system and captured the red fluorescent images (Figure 2). Finally, the lesions were biopsied or resected. In order to determine a definitive diagnosis, histopathologists from the Department of Pathology of Tsurumi University Dental Hospital performed a histological evaluation of the excised lesions. The WHO Classification [7] was used to diagnose OED.

The chromaticity x (red; C_R_) and chromaticity y (green; C_G_) of the oral lesions were measured from the fluorescence images using Eye-Scale one software (I-system, Tokyo, Japan). First, using this system, the fluorescence images were converted into chromaticity images (Figure 2), whereby C_R_ indicates redness and C_G_ expresses greenness. Subsequently, C_R_ and C_G_ on the oral lesion and normal tissue area were measured in each sample on more than 20 spots, and the average values were calculated. In addition, the C_R_ to C_G_ ratio was also calculated per the previous report [30].

### 2.4. Statistical Analysis

To estimate whether there was a significant difference between oral cancer or high-risk dysplasia and low-risk dysplasia or benign lesions, we performed an unpaired two-sided Student *t* test on the C_R_ and C_G_ and on the C_R_ to C_G_ ratio. In addition, we set each optimal cutoff value based on the receiver-operating characteristic (ROC) curve and area under the curve (AUC), which were generated using the statistical analysis software JMP (SAS Institute Inc., Tokyo, Japan).

## 3. Results

We performed ALA-PDD on 80 sites in 67 patients using a novel PDD system (Figure 1 and Figure 2; Table 1). None of the patients experienced side effects associated with ALA. Three clinicians determined whether the oral lesions emitted red fluorescence. The histopathological diagnoses of the red fluorescent areas were as follows: oral squamous cell carcinoma (OSCC), 30 of 33; carcinoma in situ (CIS), 6 of 7; and OED, severe 3 of 3, moderate 12 of 19, and mild 2 of 14 (Table 2). In contrast, for lesions that did not emit red fluorescence, the histopathological diagnoses were predominantly mild OED and benign lesions, including hyperplasia and chronic ulcers (Table 2). The sensitivity and specificity for detecting OSCC, CIS, and moderate-severe OED, called high-risk dysplasia, were 82.3% and 88.9%, respectively (Table 3). Representative images are shown below.

**Figure 3:**
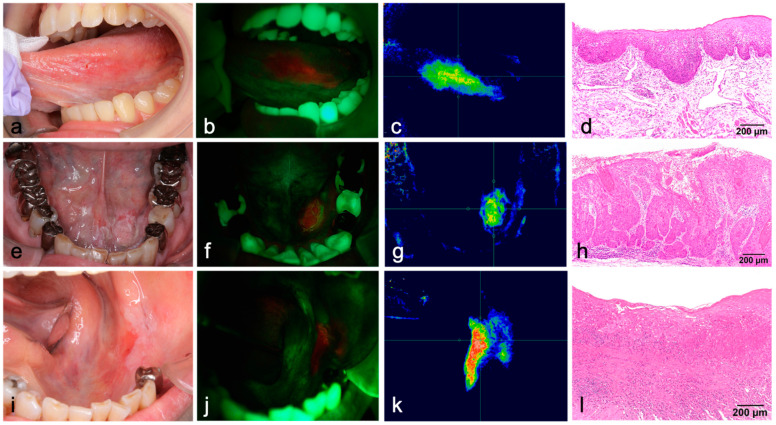
Oral findings, fluorescent images, and chromaticity of the oral lesions. (**a**–**d**) Severe OED of the tongue. (**e**–**h**) Early oral squamous cell carcinoma (OSCC) and mild OED of the oral floor. (**i**–**l**) Early OSCC of the mandibular gingiva. (**b**,**f**,**j**) Fluorescent images using ALA-PDD visualized these lesions as red fluorescence. (**c**,**g**,**k**) Converted images were used to extract redness. (**d**,**h**,**l**) histopathological findings (hematoxylin and eosin staining).

Case 1 was a 30-year-old Japanese woman with a slight erosion on the left side of the tongue, without induration (Figure 3a). ALA-PDD detected the lesion as red fluorescence, ultimately diagnosed as severe OED (Figure 3b,d). In the chromaticity image, the red fluorescence could be extracted as blue to green-red depending on the degree of redness, whereas the normal area was dark blue (Figure 3c). Case 2 was a 68-year-old Japanese man with a wide, white lesion on the oral floor (Figure 3e). The device detected red fluorescence emitted from the left side of the lesion (Figure 3f,g). This fluorescent area was diagnosed histopathologically as early invasive OSCC (Figure 3h), whereas another area with a white lesion was mild OED. If ALA-PDD had not been applied to this case, an optical biopsy site, the highly malignant area would not have been selected, leading to misdiagnosis. Case 3 was a 73-year-old Japanese woman with a white-and-red lesion on the left side of the mandible gingiva (Figure 3i). ALA-PDD detected red fluorescence emitted from the lingual side of the lesion (Figure 3j,k), and the final histopathological diagnosis was well-differentiated early OSCC (Figure 3l).

**Figure 4:**
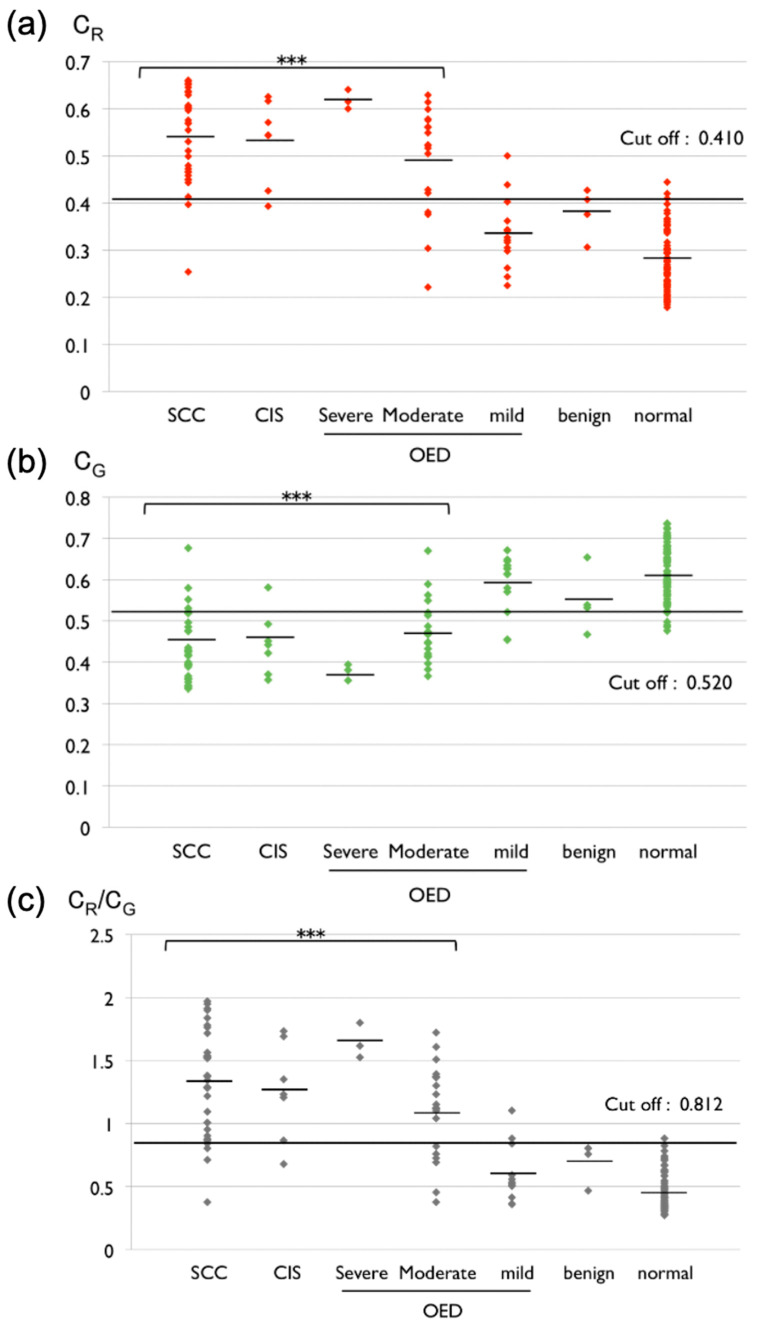
Scatter plots of the C_R_ and C_G_ and C_R_ to C_G_ ratio for different stages of oral lesions. (**a**) C_R_: chromaticity x (red). The cutoff value (0.410) indicates the borderline between OSCC, CIS, severe or moderate OED, and mild OED or benign lesions. (**b**) C_G_: chromaticity y (green). The cutoff value (0.520) differentiates between OSCC, CIS, severe and moderate OED, and mild OED and benign lesions. (**c**) C_R_/C_G_; C_R_ to C_G_ ratio (gray). The cutoff value (0.813) separates OSCC, CIS, and severe and moderate OED from mild OED and benign lesions (*** *p* < 0.001; comparison between OSCC, CIS, severe and moderate OED, and mild OED and benign lesions).

Next, we performed chromaticity analysis to quantify the fluorescence from the images (Figure 2). The chromaticity data showed that the C_R_ values for most OSCC, CIS, and high-risk dysplasia were significantly higher than those for mild OED, called low-risk dysplasia, benign lesions, and normal tissues, whereas significantly lower values of C_G_ were found for OSCC and high-risk dysplasia (*p* < 0.001; Figure 4). The cutoff values for differentiating between OSCC or high-risk dysplasia and low-risk dysplasia or benign lesions was set as 0.410 on C_R_ and 0.520 on C_G_ by the ROC curve analysis. The sensitivity and the specificity for C_R_ were 88.7% and 83.3%, respectively, whereas the sensitivity and the specificity for C_G_ were 83.3% and 83.9%, respectively (Figure 4; Table 3). To enhance the contrast between OSCC or high-risk dysplasia and low-risk dysplasia or benign lesions following the same method as outlined by the previous report [30], we attempted to estimate the C_R_ to C_G_ ratio at each oral lesion.

The C_R_ to C_G_ ratios for OSCC, CIS, and high-risk dysplasia were significantly higher than those for low-risk dysplasia and benign lesions (*p* < 0.001; Figure 4). We set the cutoff value of the C_R_ to C_G_ ratio as 0.812, and the sensitivity and specificity were 85.5% and 83.3%, respectively (Figure 4; Table 3). The qualified data were almost similar to those obtained from only ALA-PDD without qualification (Table 3). Moreover, we examined if ALA-PDD could classify high-risk dysplasia and low-risk dysplasia or benign lesions. The results demonstrated that the C_R_ and C_G_ values and the C_R_ to C_G_ ratio were significantly different between high-risk dysplasia and low-risk dysplasia or benign lesions (*p* < 0.001). We set the C_R_ and C_G_ cutoff values and the C_R_ to C_G_ ratio as 0.414, 0.520, and 0.804, respectively. The sensitivity and specificity of C_R_ were 82.8% and 83.3%, respectively; the sensitivity and specificity of C_G_ were 83.3% and 82.8%, respectively; and the sensitivity and specificity of the C_R_ to C_G_ ratio were 79.3% and 83.3%, respectively (Table 4). These results suggest that ALA-PDD could separate high-risk dysplasia from low-risk dysplasia or benign lesions with high accuracy.

## 4. Discussion

Herein, we describe the development of a handheld, user-friendly PDD system that could be used to identify oral cancers, particularly superficial, early-stage oral carcinomas and high-risk dysplasia. In addition, the data obtained from fluorescence analysis confirmed that ALA-PDD using this system might be useful for detecting and discriminating between oral cancer or high-risk dysplasia and low-risk dysplasia or benign lesions.

The conventional PDD system is an endoscope-type of procedure, which carries some difficulties in its application to the routine medical practice of the oral cavity. Indeed, when using an endoscope, an examiner must observe the oral lesions through the small tip at the end of the instrument [24,25,26,27,28,29,30]. This procedure is often complicated and requires a long observation time, leading to problems such as photobleaching which is attenuation of red fluorescence by prolonged irradiation by blue light, finally increasing false-negative rates. Thus, using an endoscope might not be suitable for observing the oral cavity. Moreover, most dental clinics and hospitals do not own an endoscope because it is not cost-effective. On the other hand, our PDD system uses a compact camera for the oral cavity, comprising a light-emitting diode light source that can irradiate only 405 nm of wavelength and specifically extract the red fluorescence (635 nm) emitted by oral lesions through specific filters. This allows for the simple acquisition of high-quality images without requiring a specialized instrument and is also affordable. Therefore, most dentists and oral surgeons can use our PDD system for diagnosing and longitudinally monitoring oral lesions as easily as taking a photograph.

Chromaticity expresses an objective specification of the quality of the color without luminescence. In this study, the red fluorescence of the oral lesions displayed on the fluorescence images obtained by ALA-PDD was appreciated as redness to qualify red fluorescence instead of measuring fluorescence intensity for the first time. Using the images converted by the software, the measure of chromaticity was easily performed. This method clearly extracted the redness on the malignant lesions from the converted images (Figure 2), especially weak red fluorescence, which examiners have difficulty judging. In addition, chromaticity analysis demonstrated the ability to qualify the redness or greenness from the images delicately. These data indicated that the C_R_ value for OSCC and high-risk dysplasia was significantly higher than for low-risk dysplasia and benign lesions (Figure 4). Interestingly, the result from the redness analysis is almost identical to the data of ALA-PDD without qualification (Table 3; Figure 4). Moreover, this result coincides with previous reports involving qualification by fluorescence spectroscopy [27,28,29,30]. From a different perspective, a significantly lower C_G_ value was found for OSCC and high-risk dysplasia (Figure 4). Chromaticity expresses color as the proportion of three primary colors: red (x), green (y), and blue (z); this mathematical formula represents x + y + z = 1. In fact, when a proportion of red (x) increases at an area, the proportions of blue and green necessarily decrease. This chromaticity principle is why the C_G_ value on the malignant lesions decreases. This analysis of C_G_ can also differentiate malignancy lesions from low-risk dysplasia and benign lesions with high sensitivity and specificity.

Furthermore, Zheng et al. and Sharwani et al. improved the accuracy of ALA-PDD by comparing the red-to-green intensity ratio between the malignant and benign lesions [28,30]. Thus, in line with these previous studies, we attempted to compare each oral lesion’s C_R_ to C_G_ ratio. Based on the C_R_ to C_G_ ratio analysis, the sensitivity and specificity were similar to those determined in the analysis of C_R_. The AUC of C_R_ and the C_R_ to C_G_ ratio indicated 0.921 and 0.909, respectively, which confirmed that ALA-PDD in combination with chromaticity analysis could represent an objective diagnostic tool for discriminating between OSCC or high-risk dysplasia and low-risk dysplasia or benign lesions with high accuracy.

The early detection of high-risk OPMDs with high-risk dysplasia, which has a high likelihood of malignant transformation, can provide patients with the best management and treatment [10]. However, there is still no consensus on the management of OED. One opinion is that high-risk dysplasia should be resected, whereas a “wait and see” policy might be applied to low-risk dysplasia [31]. On the other hand, another opinion is that all dysplastic lesions should be resected [32]. Interestingly, our results demonstrated that the boundary of the presence or absence of red fluorescence resembled the borderline between high- and low-risk dysplasia following the histopathological binary system for grading dysplasia (Table 4). Examiners might diagnose lesions emitting red fluorescence as high-risk dysplasia, whereas those with no red fluorescence may be diagnosed as low-risk dysplasia. In this regard, other light-based systems, such as autofluorescence imaging systems, can discriminate between dysplastic lesions and normal tissues but are unable to judge at this borderline [33,34]. This might be an advantage of ALA-PDD for observing oral mucosa lesions; thus, in the future, ALA-PDD might contribute to grading dysplastic lesions without the need for biopsy. Moreover, the former opinion for managing OED might be adapted if ALA-PDD were used for longitudinal monitoring of low-risk dysplasia to detect a minute transformation change. However, there are some differences in the diagnosis between the binary system and ALA-PDD. In this study, the combination of ALA-PDD and chromaticity analysis (the value of C_R_) only misclassified two of the 14 mild dysplasia cases and four of the 19 moderate OED cases (Figure 4). The binary system can reduce the variability of a diagnostic result among pathologists, but it still appears to make it difficult to judge which of the several moderate OEDs are high- or low-risk dysplasia [35]. ALA-PDD can identify alternations in cell metabolism between malignant and normal cells as red fluorescence and is likely to catch a cytological alternation that pathologists cannot obtain from fragmentary histopathological specimen sections. However, this may lead to different diagnostic results between the binary system and ALA-PDD. There are some points relating to ALA-PDD that require clarification, such as why most cases of mild OED do not emit red fluorescence despite the fact that mild OED includes atypical cells and how ALA-PDD expresses architectural alternation. These points will be addressed in future studies that will apply ALA-PDD to larger numbers of patients. ALA-PDD will provide pathologists with valuable information on oral lesions for grading dysplasia when these points are clarified.

One of the main limitations of ALA-PDD is its low specificity [25], one factor of which is thought to be a porphyrin from a large attachment of bacteria on the dorsal of a tongue and gingival plaque [25]. In the present study, to eliminate the effect of microbacteria, we performed stringent oral hygiene before ALA-PDD. Moreover, some reports have indicated that chronic inflammatory lesions, hyperplasia, radiated lesions, and papilloma emit red fluorescence [36]. In the current study, one hyperplasia emitted slight red fluorescence, while chronic ulcers didn’t; however, the reason for this has not yet been investigated. ALA-PDD has relatively low false-negative rates. The primary cause of false-negative rates is believed to be keratinization covering malignant cells and photobleaching as a technical limitation [37]. In this study, photobleaching did not affect the ALA-PDD classification at all because red fluorescence images were quickly captured by this device. In the analysis of C_R_, the false-negative rates included two of OSCC at a very early stage, one of CIS and four of moderate OED that had no keratinization, and no technical errors occurred during ALA-PDD. In addition, seven lesions had no specific histopathological features but might not have an alternation of transporters and metabolic enzymes relevant to the accumulation of PpIX. Thus, these lesions must be estimated in detail using the molecular biological techniques in the future.

## 5. Conclusions

Using our system in combination with chromaticity analysis, ALA-PDD may serve as an easy-to-use, supplementary tool for the early detection of oral cancer and OPMDs with high-risk dysplasia. This may allow for discrimination between high- and low-risk dysplasia and subsequently contribute to improved prognosis through a minimally invasive treatment and longitudinal surveillance of OPMDs.

## Figures and Tables

**Table 1:** Clinical characteristics of the patients.

**Sex**	
Male	33
Female	34
**Age**	30–92
Average	64.8
**Site**	
Tongue	51
Floor of mouth	7
Gingiva	15
Palatal	1
Buccal mucosa	6

**Table 2:** Red fluorescence regarding histopathological diagnosis.

Red Fluorescence	Histopathological Diagnosis						
	OSCC	CIS	OED			Benign	Total
			Severe	Moderate	Mild		
Positive	30	6	3	12	2	0	53
Negative	3	1	0	7	12	4	27
Total	33	7	3	19	14	4	80

OSCC: oral squamous cell carcinoma; CIS: carcinoma in situ; OED: oral epithelial dysplasia; benign: benign lesions, including chronic inflammation and hyperplasia.

**Table 3:** Comparison between ALA-PDD and the combination of ALA-PDD and chromaticity analysis for discriminating between oral cancer or high-risk dysplasia and low-risk dysplasia or benign lesions.

	PDD	C_R_	C_G_	C_R_ to C_G_
AUC	—	0.921	0.890	0.909
Significance	—	*p* < 0.001	*p* < 0.001	*p* < 0.001
Cutoff value	—	0.410	0.520	0.812
Sensitivity (%)	82.3	88.7	83.3	85.5
Specificity (%)	88.9	83.3	83.9	83.3
PPV	0.962	0.948	0.945	0.946
Accuracy	0.838	0.875	0.838	0.850

AUC: area under the curve; PPV: positive predictive value.

**Table 4:** Comparison between ALA-PDD and the combination of ALA-PDD and chromaticity analysis for discriminating between high-risk dysplasia and low-risk dysplasia or benign lesions.

	PDD	C_R_	C_G_	C_R_ to C_G_
AUC	—	0.895	0.875	0.883
Significance	—	*p* < 0.001	*p* < 0.001	*p* < 0.001
Cutoff values	—	0.414	0.520	0.804
Sensitivity (%)	72.4	82.8	83.3	79.3
Specificity (%)	88.9	83.3	82.8	83.3
PPV	0.913	0.889	0.889	0.885
Accuracy	0.787	0.830	0.830	0.809

AUC, area under the curve; PPV, positive predictive value.

## Data Availability

Data are available upon reasonable request. Requests should be sent to the corresponding author and are subject to approval.
